# Efficacy of universal preoperative decolonization with Polyhexanide in primary joint arthroplasty on surgical site infections. A multicenter before-and after-study

**DOI:** 10.1186/s13756-020-00852-0

**Published:** 2020-11-30

**Authors:** Björn Wandhoff, Christin Schröder, Ulrich Nöth, Robert Krause, Burkhard Schmidt, Stephan David, Eike-Eric Scheller, Friedrich Jahn, Michael Behnke, Petra Gastmeier, Tobias Siegfried Kramer

**Affiliations:** 1 Institute of Hygiene and Environmental Medicine, Charité-Universitätsmedizin Berlin, Hindenburgdamm 27, 12203 Berlin, Germany; 2 National Reference Center for Surveillance of Nosocomial Infections, Hindenburgdamm 27, 12203 Berlin, Germany; 3 Evangelisches Waldkrankenhaus Spandau, Stadtrandstraße, 555 Berlin, Germany; 4 LADR MVZ GmbH Neuruppin, Zur Mesche 20, 16816 Neuruppin, Germany; 5 Oberlinklinik-Orthopädische Fachklinik, Rudolf-Breitscheid-Straße 24, 14482 Potsdam, Germany; 6 Evangelisches Krankenhaus Paul Gerhardt Stift, Paul-Gerhardt-Straße 42-45, 06886 Lutherstadt Wittenberg, Germany; 7 Evangelisches Krankenhaus Hubertus, Spanische Allee 10-14, 14129 Berlin, Germany; 8 Evangelische Elisabeth Klinik, Lützowstraße 26, 10785 Berlin, Germany

**Keywords:** Surgical site infection, Periprosthetic joint infection, Decolonization, Polyhexanide, Total joint arthroplasty

## Abstract

**Background:**

Surgical site infections (SSI) are rare but severe complications after total joint arthroplasty (TJA). Decolonization measures prior to elective orthopedic surgeries have shown to reduce the risk of SSI with *Staphylococcus aureus* (*S. aureus*).

**Objective:**

To determine the efficacy of universal decolonization with Polyhexanide on SSI rates with a focus on *Staphylococcus aureus* in patients with TJA.

**Methods:**

Patients scheduled for elective hip or knee TJA in 5 participating certified orthopedic centers were included between 2015 and 2018 into this before and after study. Data on patients, surgeries and infections were prospectively collected. CDC-criteria were used to define and categorize Infections within 90 days after surgery. From January 2017 on, patients received decolonization sets containing Polyhexanide. Patients performed a 5 day decolonization regimen starting 4 days prior to surgery which included wipes, nasal decontamination and oral solution.

**Results:**

Thirteen thousand, three hundred fifteen patients received TJA. During intervention 4437 decolonization sets were distributed among 7175 patients.

Overall SSI rates increased from 0.68 /100 surgeries to 0.91/ 100 surgeries after implementation of the intervention (IRR 1.32; 95% CI 0.90–1.96). Time series analysis identified an increasing trend of SSI prior to the intervention. After implementation overall SSI rates plateaued. Regression analysis revealed surgery during intervention period to be an independent risk factor for developing a SSI (OR 1.34; 95%CI 1.18–1.53).

Initial SSI rates due to *S. aureus* were 0.24/100 surgeries and decreased to 0.14/100 surgeries (IRR 0.57; 95% CI 0.25–1.22) after introduction of decolonization. Regression analysis revealed surgery during intervention period to be an independent protective factor for developing a SSI with *S. aureus* (OR 0.57, 95% CI: 0.33–0.99).

Overall deep *S. aureus* SSI decreased significantly from 0.22/100 surgeries to 0.00/100 surgeries in patients adherent to protocol (IRR 0.00, 95% CI 0.00–.85).

**Conclusion:**

Universal decolonization with Polyhexanide did not reduce overall surgical site infections, but was effective in reducing *Staphylococcus aureus* - surgical site infections following elective joint arthroplasty. Polyhexanide could extend the list of alternatives to already established decolonization strategies.

***Trial registration*:**

The trial was registered at the German Registry for clinical studies www.drks.de (DRKS00011505).

## Background

Surgical site infections (SSI) are a rare but severe complication of total joint arthroplasty (TJA) [1]. Their absolute numbers are expected to increase due to an increasing number of implantations especially of hip and knee joint arthroplasty [2].

Several risk factors such as obesity, gender and duration of surgery have been identified as risk factors for developing a SSI [3]. In particular being a carrier of *Staphylococcus aureus* (*S. aureus*) has been well described as an independent risk factor for *S. aureus* SSI [4, 5]. Most early postoperative SSI are caused by previous *S. aureus* colonizing the patient [6, 7].

- To identify and decolonize *S. aureus* carriers with Chlorhexidine and Mupirocin prior to TJA is an effective measure to decrease SSI [6, 8]. This targeted decolonization strategy was recently adopted by the WHO [9] and the CDC [10] as well as being in the German national recommendations for prevention of SSI [11]. Implementation of additional screening for *S. aureus* prior to surgery can prove difficult in some settings and can be of limited sensitivity [12]. Universal decolonization of all patients for TJA prior to surgery was shown to reduce SSI and further improves cost effectiveness when compared to targeted decolonization [13]. Resistance against Mupirocin and Chlorhexidine has increasingly been reported in the recent past [14–16]. This seems to apply not only for *S. aureus*, but especially for Coagulase negative Staphylococci (ConS) after introduction of universal decolonization in patients for TJA. ConS are the most frequent cause of low grade periprosthetic joint infections. High rates of resistance in ConS could potentially decrease effectiveness. Furthermore severe adverse events have been reported with the use of Chlorhexidine in the past [17]. Therefore, alternative substances and decolonization strategies have been evaluated or are currently being used [18, 19]. Polyhexanide is a safe antiseptic substance, which is widely used for wound disinfection [20–22]. A randomized controlled trial showed no superiority for the decolonization of MRSA carriers when compared to placebo [21]. Some reports suggest that it is at least as effective as Chlorhexidine [23, 24]. Despite its widespread use, resistance against Polyhexanide has not yet been described in *S. aureus* [15] or other pathogens. For *E. coli* differences in minimal inhibitory concentration for Polyhexanide have been described among clinical strains [25].
- Thus the objective of this study was to identify the effect on SSI especially those caused by *S. aureus* after implementation of a universal preoperative decolonization utilizing Polyhexanide in patients for hip and knee joint arthroplasty.

## Methods

### Setting, study design and data collection

Six certified TJA centers in orthopedic and traumatology departments in the German federal states of Berlin, Brandenburg and Sachsen-Anhalt agreed to participate in this before and after study. The centers vary in the number of arthroplasty surgeries performed during the study period. The number of surgeries performed during the study period ate the centers range from 172 to 3178 representing a large variety. Due to organizational reasons one center was unable to give patient instructions and distribute sets to patients on their preoperative visit within 4 weeks after beginning of the intervention period. This center was excluded from the study and all of the analysis. Between January 2015 and December 2018, data on patients with primary hip and knee arthroplasty were collected prospectively, following the protocols of the SSI module of the national hospital infection surveillance system (OP-KISS). Protocols have been described in detail elsewhere [7, 26]. SSIs were prospectively recorded according to CDC criteria. These specify a surveillance period of 90 days for SSI. Cases of SSI were either documented prior to discharge or upon readmission. No explicit post discharge surveillance on SSI was performed during the study. But cases of SSI were detected upon readmission. Infections were either classified as superficial or deep. The group of deep infections consisted of deep SSI and SSI of the organ space. Pathogens causing the infection were recorded as *S. aureus* or grouped into coagulase negative Staphylococci (ConS), *Streptococcus spp.*, *Enterococcus spp.*, *Cutibacterium spp.*, gram negative rods and “Other pathogens”. In addition Infections without microbiological confirmation were documented at the surgeons’ discretion.

Documentation of infection was prospectively performed by trained, specialized infection control staff of the individual centers. Additional variables collected for each patient that received a tracer surgery were gender, age, ASA-score, and duration of surgery.

### Intervention

Starting January 1st, 2017, all patients with elective joint arthroplasty were offered use a set consisting of wipes containing Polyhexanide (0.11% Poliaminopropyl Biguanid; once daily), Polyhexanide nasal ointment (three times daily) and Polyhexanide oral solution (three times daily). On their preoperative visit, patients with elective hip and knee arthroplasties received a demonstration on how to use the set according to the manufacturer’s recommendations. Patients were instructed to use the wipes after showering without the use of any cosmetic products, creams, or lotions during the decolonization period. In most cases instructions were given by treating surgeons. Additional printed instructions were included in the set. Patients received the set at the preoperative visit. They were asked to use the set for 5 days starting 4 days prior to surgery. Five day decolonization for *S. aureus* is commonly used [8]. These patients were also asked to fill out a survey, and to report on their compliance and any adverse events. For analysis three cohorts were created:

1. Control group; All patients with surgery prior to intervention (2015/2016)
2. Intervention group; All patients with surgery during intervention period (2017/2018)
3. Adherent to protocol subgroup; patients that gave consent and used the decolonization set during intervention period (2017/2018)

For additional time series analysis (Additional file 4: Figure 1) the intervention group was divided into adherent to protocol (adherent) and patients with unknown adherence (other).

No other changes or adaptions to the infection prevention strategies were implemented during the study period. No structured antiseptic decolonization or showering strategy was established prior to this study. All centers applied perioperative antibiotic prophylaxis within thirty to sixty minutes prior to incision with an intravenous 1st/2nd generation cephalosporin or intravenous clindamycin in patients with a type 1 penicillin allergy or MRSA colonization. Redosing of the antibiotic is performed at ninety minutes if the duration of surgery exceeds this duration. All theatres in the participating centers use a laminar or turbulent HEPA filtrated airflow. They comply with national recommendations.

Severe adverse events were defined as reversible or irreversible medical conditions that occurred in conjunction with the intervention and needed medical treatment (ea. anaphylaxis; severe exanthema). Adverse events were defined as reversible conditions that occurred in conjunction with the intervention and needed no medical treatment (e.g. xerosis cutis, dermatitis).

### Ethics

The ethics committee at Charité Universitätsmedizin Berlin approved the study (Internal Key: EA4/124/16). The trial was registered at the German Registry for clinical studies www.drks.de (DRKS00011505).

### Statistical methods

Overall 4577 patients needed to receive the intervention according to our sample size calculation based on SSI rates of the centers from 2015 with an estimated reduction of 40% SSI and with a power of 80% and an alpha of 0.05.

The median and the interquartile ranges (IQR) were calculated for continuous parameters; number and percentage were calculated for binary parameters.

For univariate comparison between baseline period and intervention period as well as baseline period and patients adherent to protocol incidence rate ratios with 95%-CI and mid-*p*-values were calculated (R package epitools V 0.5–10 were used for calculation).

Multivariable models were created to investigate the effect of the intervention on SSI and *S.aureus* SSI in patients with elective TJA (total hip arthroplasty; total knee arthroplasty). Logistic regression using generalized linear mixed effect models with outcome were utilized in order to estimate overall SSI and *S. aureus*-SSI. Independent risk factors in the model were time of surgery in the intervention period (yes/no), age under median (yes/no), duration of surgery under 75%-quantile (yes/no), ASA Score equal or less than 2 (yes/no), and sex (male/female). Taking into account the cluster effect, the model was estimated with an exchangeable correlation structure for center. Time series analysis was carried out similarly. Two effects for time trend were additionally included in analysis to the effect of intervention – time trend for whole time series and time trend for period of intervention. Odds ratios with 95%- confidence interval were calculated for both models.

A *p*-value of < 0.05 was considered significant. All analyses were performed using R [27] and SAS. The R package ggplot2 (V 3.1.1) was used for graphics [28].

## Results

During the study period 13,315 patients received replacement of their primary joint (7, 820 elective hip prosthesis; 5495 elective knee prosthesis). 7, 175 patients received surgery after implementation of universal decolonization. 4, 377 sets (61%) were distributed to patients at the participating centers for universal decolonization. Compliance with the intervention protocol was confirmable for 1866 patients who had given their explicit consent and answered the survey (Table 1). No severe adverse events were reported, but *n* = 251 patients reported mild adverse events of their skin (*n* = 150), their mouth/pharynx (*n* = 74) or nose (*n* = 42).

**Table 1:** Characteristics of patients undergoing elective total joint arthroplasty in the preintervention and intervention period

	Preintervention period	Intervention period		Total
	Control	Adherent to protocol (subgroup)	Intervention (total)	
Total joint arthroplasty	6140	1866	7175	13,315
Hip (%)	3645 (59.4%)	1041 (55.8%)	4175 (58.2%)	7820 (58.7%)
Knee (%)	2495 (40.6%)	825 (44.2%)	3000 (41.8%)	5495 (41.3%)
Female (%)	3814 (62.1%)	1150 (61.6%)	4365 (60.8%)	8179 (61.4%)
Age (Median [IQR])	72 [63, 77]	70 [63, 77]	71 [63, 78]	71 [63, 77]
ASA Score < 3 (%)	4319 (70.3%)	1327 (71.1%)	4987 (69.5%)	9306 (69.9%)
Duration of surgery >Median % [IQR]	65 [52, 80]	60 [49, 74]	62 [50, 75]	63 [51, 77]
Days until SSI Median [IQR]	28.50 [19.25–50.25]	15 [10–39]	20 [15–28]	23 [15–41]

*IQR* interquartile range

**Fig. 1:**
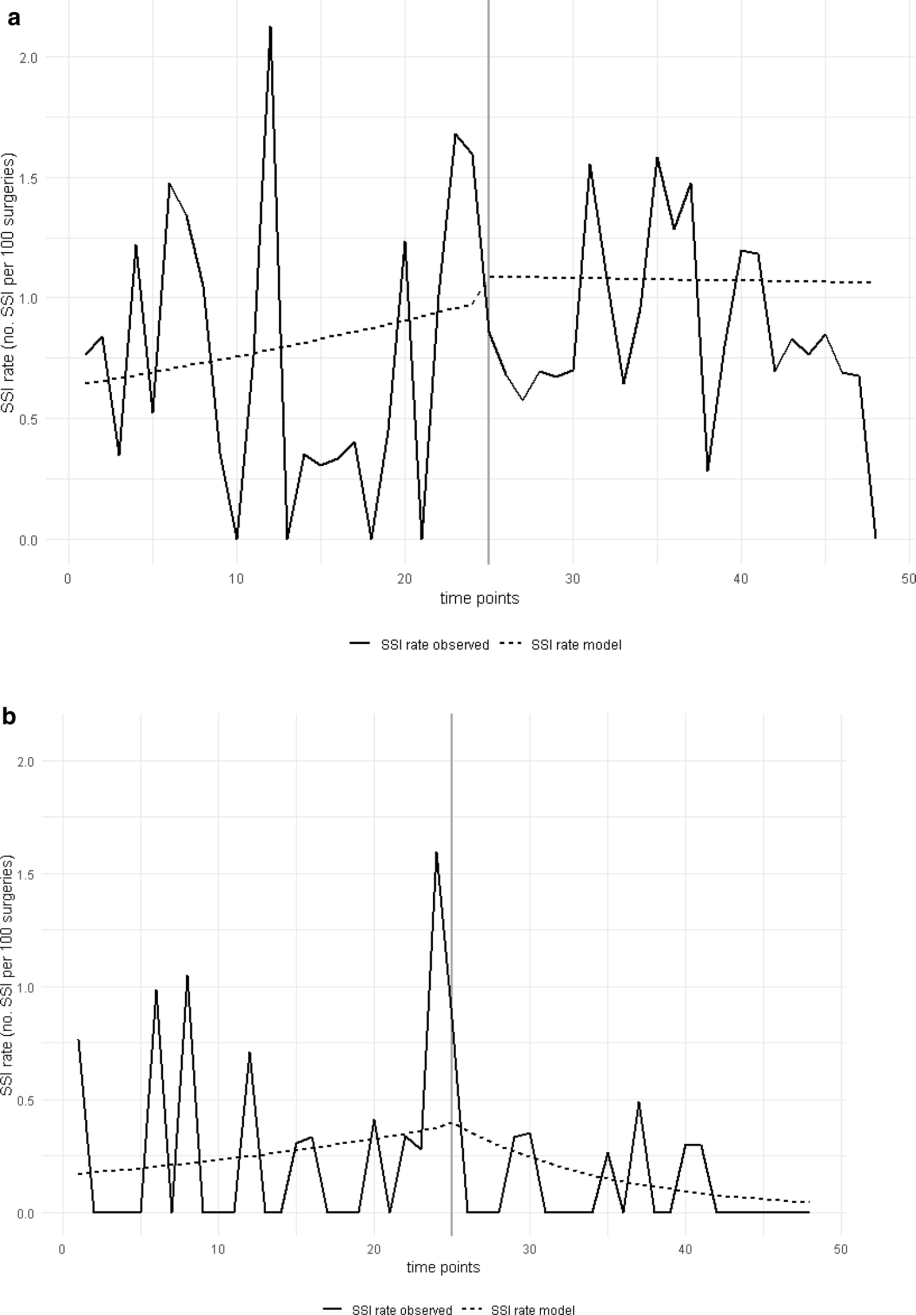
**a** Rate of observed and modelled overall Surgical Site Infection rate (SSIR) and **b** *Staphylococcus aureus* Surgical Site Infection rate (*S*. *aureus* -SSIR) by month of surgery in the period prior to (0–24) and after implementation of the intervention (25–48)

**Fig. 2:**
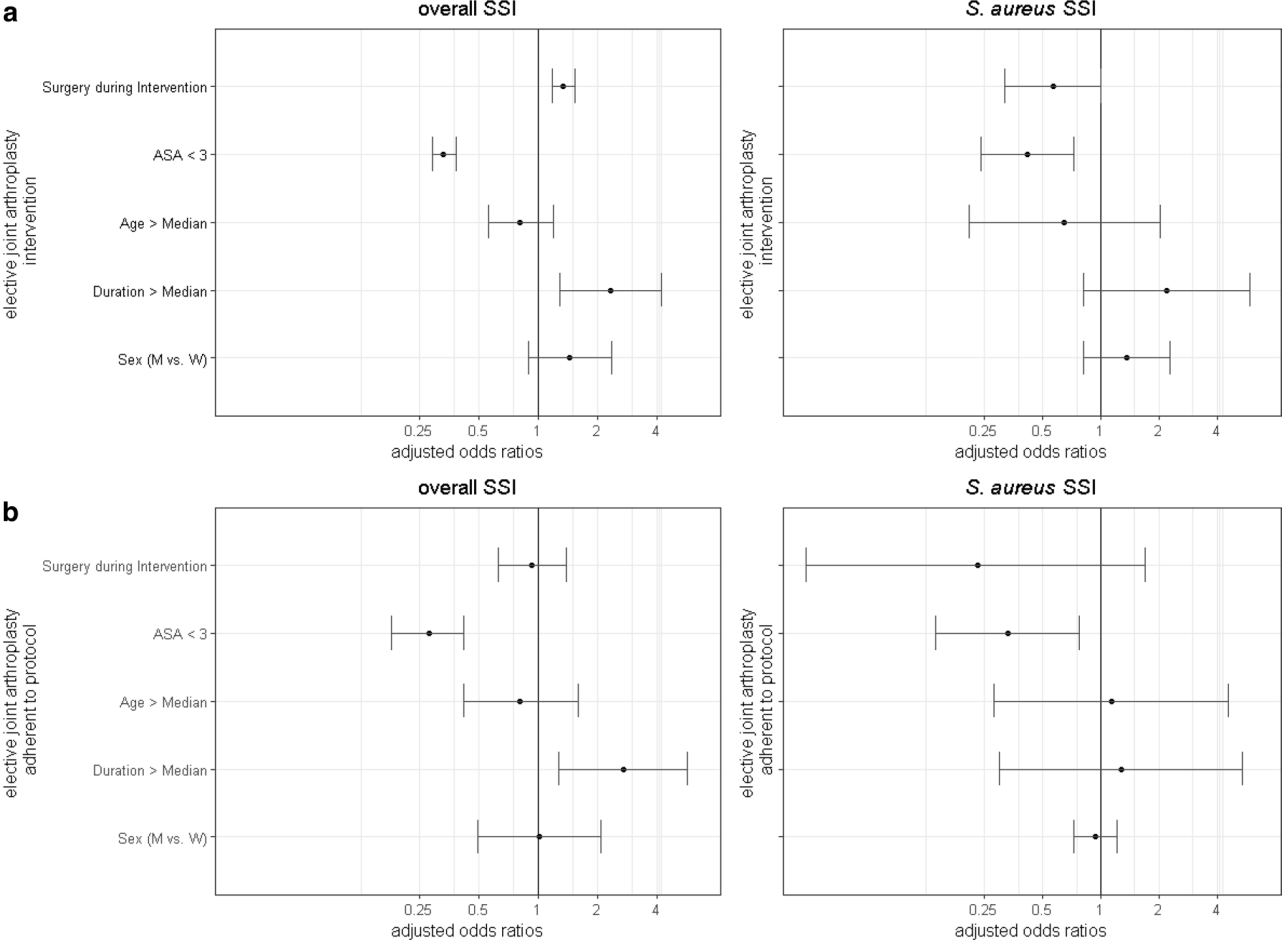
Results of multivariable regression analysis with the endpoints overall SSI and *S. aureus* SSI (**a**) in all patients with elective joint arthroplasty and **b** in patients adherent to protocol

Overall SSI rates were 0.68/100 surgeries in the control group and 0.91/100 surgeries in the intervention group after implementation of the decolonization (IRR 1.32, 95% CI .90–1.96; Table 2). They were 0.59 /100 surgeries in the subgroup adherent to protocol (IRR 0.87, 95% CI 0.42–1.64). These changes were statistically not significant. Time series analysis identified an increasing trend of SSI prior to the intervention. After implementation of the decolonization SSI rates plateaued in the intervention group (Fig. 1), but decreased in the subgroup adherent to protocol (Additional file 5: Figure 2). Regression analysis revealed surgery during intervention period to be an independent risk factor for developing a SSI (OR 1.34; 95%CI 1.18–1.53; Fig. 2).

**Table 2:** Surgical site infections (SSI) in the study cohort

	Control N (SSIR)	Adherent to protocol N (SSIR)	Intervention N (SSIR)	Total N (SSIR)		
					Control vs. Adherent to protocol IRR [95%CI], *p*-value	Control vs. Intervention IRR [95%CI], *p*-value
**Overall**						
Total infections	42 (0.68)	11 (0.59)	65 (0.91)	107 (0.80)	0.87 [0.42, 1.64] 0.68	1.32 [0.90, 1.96] 0.15
Hip infections	29 (0.80)	10 (0.96)	49 (1.17)	78 (1.00)	1.22 [0.56, 2.43] 0.60	1.47 [0.94, 2.36] 0.09
Knee infections	13 (0.52)	1 (0.12)	16 (0.53)	29 (0.53)	0.26 [0.01, 1.33] 0.12	1.02 [0.49, 2.18] 0.95
***S. aureus***						
Infections due to *S. aureus*	15 (0.24)	1 (0.05)	10 (0.14)	25 (0.19)	0.25 [0.01, 1.23] 0.10	0.57 [0.25, 1.27] 0.17
Hip *S. aureus* infections	10 (0.27)	1 (0.10)	6 (0.14)	16 (0.20)	0.40 [0.02, 2.09] 0.32	0.52 [0.18, 1.45] 0.22
Knee *S. aureus* infections	5 (0.20)	0 (0.00)	4 (0.13)	9 (0.16)	0 [0.00, 2.48] 0.24	0.67 [0.16, 2.63] 0.56

*SSIR* Surgical site infection rate, Infections/100surgeries, *IRR* Incidence Rate Ration

In patients with elective knee arthroplasty that were adherent to protocol, surgery during intervention was an independent protective factor in developing a SSI overall (Additional file 5: Figure 2).

After implementation of decolonization SSI caused by *S. aureus* decreased from 0.24/ 100 surgeries to 0.14/ 100 surgeries (IRR 0.57, 95% CI 0.25–1.27) in the intervention group and to 0.05/ 100 surgeries (IRR 0.25, 95% CI 0.01–1.23) in the subgroup adherent to protocol. These changes were statistically not significant. Time series analysis shows a continuous increase in *S. aureus* SSI rates prior to implementation of the intervention, followed by a decrease afterwards (Fig. 1). Multivariable regression analysis of SSI caused by *S. aureus* in elective total joint arthroplasty did identify surgery during intervention as an independent protective factor for developing an SSI caused by *S. aureus* (OR .57, 95% CI: 0.33–0.99). Overall Deep *S. aureus* SSI decreased significantly from 0.22/100 surgeries to 0.00/100 surgeries in patients adherent to protocol (IRR 0.00, 95% CI 0.00–0.85; Additional file 1: Table 1).

An ASA score of less than three was an additional protective factors for development of SSI in general (OR 0.33, 95% CI: 0.29–0.38). Duration of surgery above the median was identified as a risk factor for development of SSI (OR 2.34, 95%CI 1.28–4.25, Fig. 2).

## Discussion

Our study evaluated the effect of implementing universal decolonization with Polyhexanide in joint arthroplasty on SSI in centers with an average SSI-rate for TJA [29]. Overall, SSI rates increased in patients with TJA after implementation of the intervention but not in those that were adherent to protocol. SSI due to *S. aureus* decreased in the intervention group as well as the subgroup that was adherent to protocol.

Differences in patient characteristics recorded were neither identified among patients in the pre- and intervention phase nor in patients that were adherent to protocol.

Overall SSI rates increased after the implementation of the intervention. This increase was solely due to SSI occurring in patients with total hip arthroplasty, but not in those receiving total knee arthroplasty. In addition this increase did not occur in patients with total hip arthroplasty adherent to protocol. Time series analysis shows an increase of SSI rates prior to introduction of the intervention, which stabilized in the intervention phase. In addition no single group of pathogens or species, which might have replaced the local skins microbiome after the intervention, was identified to be the cause of the increase in SSI (Additional file 1: Table 1). During the intervention phase more infections caused by difficult to culture bacteria such as *Cutibacterium spp.* and without microbiological confirmation were documented. In addition median time to SSI after surgery was shorter during the intervention period. A real change of the median time to onset of infection of 20 days is possible, but less likely than earlier diagnosis of infection. These differences were not statistically significant. However we could speculate that these observations are potentially based on changed perception regarding SSI that lead to earlier diagnostic and therapeutic intervention in patients with SSI after implantation in the participating centers. These observations suggest that the increase of SSI overall was presumably not caused by the intervention.

Only few studies focused on the effect of overall SSI, but mostly reported *S. aureus* SSI as their primary outcome. These studies showed reduction of SSI for decolonization strategies using different substances [30, 31]. Bode et al. were able to show reduced *S. aureus* SSI in colonized patients undergoing orthopedic surgery after using their decolonization protocol consisting of Chlorhexidine and Mupirocin (1 infection in 87 patients) when compared to control (4 infections in 81 patients) [6]. Schweizer et al. used a targeted decolonization strategy with Chlorhexidine and Mupirocin in patients undergoing THA decreasing deep complex *S. aureus* SSI from 0.4/100 surgeries to 0.2/100 surgeries [8]. Another randomized controlled trial neither found a reduction of SSI in patients not colonized nor in patients colonized with *S. aureus* [32].

Regression analysis showed that receiving elective surgery with an ASA score of less than three was identified as independent protective factors [33], confirming known non modifiable risk factors on patient’s site. Increased length of surgery is a well-established risk factor for developing SSI, those findings support the trust in our models [34, 35].

While overall 4800 sets were available to the centers, the ratio of distributed sets (*n* = 4377) to patients (*n* = 7633) show that many patients did not receive an intervention kit. Some patients that were offered to receive a set declined or were not able to use the kit due to physical impairment. Even though not in line with the study protocol, it is possible that temporary selection processes by the health care workers and surgeons at individual centers facilitated the high number of patients without sets in the intervention group. Unfortunately we are not able to identify beyond a doubt which patients that did not give their informed consent or did not receive a set. In addition 433 sets expired during the Intervention and were therefore not distributed. All of these factors potentially reduced the interventions full effect, but reflect a real life clinical setting.

Due to the 90-day surveillance period, SSI in this study mostly represents early postoperative periprosthetic joint infections. SSI due to *S. aureus* are most frequently found in early postoperative phase [36]. However effects on low grade infections that usually are diagnosed up to 2 years after surgery were beyond the scope of our surveillance system.

There are further limitations to our study, which need to be addressed. i) As a result of the study design, prospectively collected SSI cases are prone to certain influences such as change in surgical approach or diagnostic procedures that occurred during the study period. In center 2 an interdisciplinary SSI team was implemented in 2017 along with a rapid recovery program for all hip and knee arthroplasty, which changed the centers surgical management (Additional file 2: Table 2). ii) The intervention was not blinded. Therefore, identification of SSI might have been influenced despite preexisting case definitions. iii) Changes of patient’s characteristics during the study period are unlikely. But they cannot be ruled out since well described risk factors such as BMI are not routinely recorded in the surveillance system. In addition patients adherent to interventions generally do better and have improved outcome iv) We only detected *S. aureus*-SSI within the first 90 days after surgery. The majority of SSI in TJA occurs within 2 years after implantation. But these are less frequently due *S. aureus*. v) According to our calculations, we did not reach necessary sample size. Therefore this study was underpowered and interpretations of results require careful assessment.

## Conclusion

Overall SSI did not decrease after implementation of the intervention. Universal decolonization with Polyhexanide was effective in reducing *Staphylococcus aureus* - surgical site infections following elective joint arthroplasty. This could potentially establish preoperative decolonization with Polyhexanide as an alternative to already established substances. Future prospective comparative studies are needed to evaluate the non-inferiority of this decolonization strategy in patients with elective joint arthroplasty.

## Supplementary Information

**Additional file 1.** Supplement Table 1. Overview of Surgical Site Infections (SSI)

**Additional file 2**. Supplement Table 2. Overview of tracer surgeries and Surgical Site Infections in the individual participating centers

**Additional file 3**. Supplement Table 3. Orion checklist

**Additional file 4**. Supplement Figure 1. Rate of observed and modelled overall Surgical Site Infection rate (SSIR) by month of surgery in the period prior to (0–24) and after implementation of the intervention (25–48) in patients adherent to protocol and unknown adherence (other)

**Additional file 5**. Supplement Figure 2. Results of multivariable regression analysis with the endpoints overall and *S. aureus* SSI a) in all patients with elective hip arthroplasty b) in patients with elective hip arthroplasty adherent to protocol c) in all patients with elective knee arthroplasty d) in patients with elective knee arthroplasty adherent to protocol

## Data Availability

The datasets used and analyzed during the current study are available from the corresponding author on reasonable request.

## Abbreviations

95%CI: 95% confidence interval
ASA: American Society of Anesthesiologists
ConS: Coagulase negative Staphylococci
IQR: Interquartile range
IRR: Incidence rate ration
OR: Odds Ratio
*S. aureus*: *Staphylococcus aureus*
SSI: Surgical Site Infection
SSIR: Surgical Site Infection Rate; arthroplasty
TJA: Total joint arthroplasty
