# Supplementary material for: Efficacy of universal preoperative decolonization with Polyhexanide in primary joint arthroplasty on surgical site infections. A multicenter before-and after-study
Source: Antimicrob Resist Infect Control. 2020 Nov 30;9:188. doi: 10.1186/s13756-020-00852-0 (PMC7708093; doi:10.1186/s13756-020-00852-0)
Supplement: Supplementary file 1 — Additional file 1. Supplement Table 1. Overview of Surgical Site Infections (SSI) [file 13756_2020_852_MOESM1_ESM.docx]

Supplement table 1: Overview of Surgical Site Infections (SSI)

|  | Control  N (SSIR) | Adherent to protocol  N (SSIR) | Intervention  N (SSIR) | Total  N SSIR) |  | |
| --- | --- | --- | --- | --- | --- | --- |
|  |  |  |  |  | Control vs. Adherent to protocol  IRR [95%CI], p-value | Control vs. Intervention  IRR [95%CI], p-value |
|  | | | | | | |
| Superficial SSI | 9 (0.15) | 3 (0.16) | 11 (0.15) | 20 (0.15) | 1.13 [0.24, 3.88]  0.85 | 1.04 [0.43, 2.62]  0.93 |
| Deep SSI | 33 (0.53) | 8 (0.43) | 54 (0.75) | 87 (0.66) | 0.81 [0.35, 1.67]  0.59 | 1.40 [0.91, 2.18]  0.13 |
|  | | | | | | |
| *Staphylococcus aureus* | 15 (0.24) | 1 (0.05) | 10 (0.14) | 25 (0.19) | 0.25 [0.01, 1.23], 0.10 | 0.57 [0.25, 1.27], 0.17 |
| Superficial *S. aureus* SSI | 2 (0.03) | 1 (0.05) | 3 (0.05) | 12 (0.20) | 1.75 [0.06, 21.63]  0.69 | 1.25 [0.19, 10.79]  0.81 |
| Deep *S. aureus* SSI | 13 (0.22) | 0 (0.00) | 7 (0.12) | 8 (0.13) | 0.00 [0.00, 0.85]  0.03 | 0.47 [0.17, 1.15]  0.10 |
|  | | | | | | |
| ConS | 13 (0.21) | 3 (0.16) | 16 (0.22) | 29 (0.22) | 0.79 [0.17, 2.48], 0.71 | 1.05 [0.50, 2.24], 0.89 |
| *Streptococcus spp.* | 1 (0.02) | 1 (0.05) | 7 (0.1) | 8 (0.06) | 3.29 [0.08, 128.33], 0.47 | 5.35 [0.93, 135.50], 0.06 |
| *Enterococcus spp.* | 2 (0.03) | 1 (0.05) | 3 (0.04) | 5 (0.04) | 1.75 [0.06, 21.63],  0.69 | 1.25 [0.19, 10.79], 0.81 |
| gramnegative rods | 5 (0.08) | 2 (0.11) | 9 (0.13) | 14 (0.11) | 1.37 [0.18, 6.67]  0.72 | 1.52 [0.52, 5.07]  0.45 |
| *Cutibacterium spp.* | 0 (0.00) | 1 (0.05) | 8 (0.08) | 8 (0.08) | n.e. | n.e. |
| Other pathogens | 5 (0.08) | 1 (0.05) | 6 (0.08) | 11 (0.08) | 0.73 [0.03, 4.75], 0.47 | 1.02 [0.30, 3.66], 0.97 |
| without microbiological confirmation | 1 (0.02) | 1 (0.05) | 6 (0.08) | 7 (0.05) | 3.29 [0.08, 128.33], 0.47 | 4.60 [0.76, 118.59], 0.10 |

Surgical site infection rate (SSIR): Infections/100 surgeries; Incidence Rate Ratio (IRR)

; Coagulase negative Staphylococci (ConS)
