# Supplementary material for: Efficacy of universal preoperative decolonization with Polyhexanide in primary joint arthroplasty on surgical site infections. A multicenter before-and after-study
Source: Antimicrob Resist Infect Control. 2020 Nov 30;9:188. doi: 10.1186/s13756-020-00852-0 (PMC7708093; doi:10.1186/s13756-020-00852-0)
Supplement: Supplementary file 2 — Additional file 2. Supplement Table 2. Overview of tracer surgeries and Surgical Site Infections in the individual participating centers [file 13756_2020_852_MOESM2_ESM.docx]

Supplement table 2: Overview of tracer surgeries and Surgical Site Infections in the individual participating centers

|  | | **elective hip joint arthroplasty** | | | | | **elective knee joint arthroplasty** | | | | |
| --- | --- | --- | --- | --- | --- | --- | --- | --- | --- | --- | --- |
|  |  | Center | | | | | Center | | | | |
|  |  | 1 | 2 | 3 | 4 | 5 | 1 | 2 | 3 | 4 | 5 |
| Surgeries | Control | 70 | 1223 | 1867 | 282 | 203 | 102 | 768 | 1311 | 0 | 314 |
|  | Adherent to protocol | 32 | 417 | 483 | 71 | 38 | 31 | 240 | 409 | 48 | 97 |
|  | Intervention | 173 | 1658 | 1983 | 183 | 178 | 233 | 999 | 1326 | 106 | 336 |
|  | Total | 243 | 2881 | 3850 | 465 | 381 | 335 | 1767 | 2637 | 106 | 650 |
| Duration of surgery in minutes (median) | Control | 83.09 | 63.79 | 59.55 | 66.48 | 59.51 | 73.30 | 91.41 | 71.17 | 0 | 67.92 |
|  | Adherent to protocol | 80.56 | 58.60 | 54.57 | 63.70 | 51.68 | 68.10 | 81.85 | 67.71 | 73.50 | 51.36 |
|  | Intervention | 87.46 | 60.19 | 56.29 | 60.83 | 53.52 | 70.71 | 81.85 | 68.09 | 72.46 | 55.59 |
|  | Total | 86.20 | 61.72 | 57.87 | 64.25 | 56.71 | 71.50 | 86.00 | 69.62 | 72.46 | 64.54 |
| Infections | | | | | | | | | | | |
| SSI | Control | 0 | 15 | 11 | 2 | 1 | 0 | 4 | 7 | 0 | 2 |
|  | Adherent to protocol | 0 | 4 | 4 | 2 | 0 | 0 | 0 | 1 | 0 | 0 |
|  | Intervention | 2 | 26 | 14 | 4 | 3 | 1 | 6 | 7 | 1 | 1 |
|  | Total | 2 | 41 | 25 | 6 | 4 | 1 | 10 | 14 | 1 | 3 |
| SSI/ 100 surgeries | Control | 0.00 | 1.23 | 0.59 | 0.71 | 0.49 | 0.00 | 0.52 | 0.53 | - | 0.64 |
|  | Adherent to protocol | 0.00 | 0.96 | 0.83 | 2.82 | 0.00 | 0.00 | 0.00 | 0.24 | 0.00 | 0.00 |
|  | Intervention | 1.16 | 1.57 | 0.71 | 2.19 | 1.69 | 0.43 | 0.60 | 0.53 | 0.94 | 0.30 |
|  | Total | 0.82 | 1.42 | 0.65 | 1.29 | 1.05 | 0.30 | 0.57 | 0.53 | 0.94 | 0.46 |
|  | | | | | | | | | | | |
| *S. aureus* SSI | Control | 0 | 5 | 2 | 2 | 1 | 0 | 1 | 3 | 0 | 1 |
|  | Adherent to protocol | 0 | 0 | 0 | 1 | 0 | 0 | 0 | 0 | 0 | 0 |
|  | Intervention | 1 | 2 | 1 | 2 | 0 | 1 | 1 | 2 | 0 | 0 |
|  | Total | 1 | 7 | 3 | 4 | 1 | 1 | 2 | 5 | 0 | 1 |
| *S. aureus* SSI / 100 surgeries | Control | 0.00 | 0.41 | 0.11 | 0.71 | 0.49 | 0.00 | 0.13 | 0.23 | - | 0.32 |
|  | Adherent to protocol | 0.00 | 0.00 | 0.00 | 1.41 | 0.00 | 0.00 | 0.00 | 0.00 | 0.00 | 0.00 |
|  | Intervention | 0.58 | 0.12 | 0.05 | 1.09 | 0.00 | 0.43 | 0.10 | 0.15 | 0.00 | 0.00 |
|  | Total | 0.41 | 0.24 | 0.08 | 0.86 | 0.26 | 0.30 | 0.11 | 0.19 | 0.00 | 0.15 |
