# Supplementary material for: Efficacy of universal preoperative decolonization with Polyhexanide in primary joint arthroplasty on surgical site infections. A multicenter before-and after-study
Source: Antimicrob Resist Infect Control. 2020 Nov 30;9:188. doi: 10.1186/s13756-020-00852-0 (PMC7708093; doi:10.1186/s13756-020-00852-0)
Supplement: Supplementary file 3 — Additional file 3. Supplement Table 3. Orion checklist [file 13756_2020_852_MOESM3_ESM.docx]

Supplement table 3: Orion checklist

| **ORION Checklist of items to include when reporting an outbreak or intervention study of a nosocomial organism Item Number** | **Descriptor** | | |
| --- | --- | --- | --- |
| **Title & Abstract** | 1 | Title page; Lines 10-14 | Description of paper as outbreak report or intervention study. Design of intervention study (eg Randomised Controlled Trial , Cluster Randomised Controlled Trial, Interrupted Time Series, Cohort study etc). Brief description of intervention and main outcomes. |
| **Introduction**  Background | 2 | Lines 39-46 | Scientific and/or local clinical background and rationale.  Description of organism as epidemic, endemic or epidemic becoming endemic. |
| Type of paper | 3 | Title | Description of paper as Intervention study or an Outbreak Report.  If an outbreak report, report the number of outbreaks. |
| Dates | 4 | Lines 10, 74,89 | Start and finish dates of the study or report. |
| Objectives | 5 | Lines 62-64 | Objectives for outbreak reports. Hypotheses for intervention studies |
| **Methods**  Design | 6 | Lines 10-11  Lines 67-68 | Study design. Use of EPOC classification recommended (RCT or CRCT, CBA, or ITS)  Whether study was retrospective, prospective or ambidirectional.  Whether decision to report or intervene was prompted by any outcome data.  Whether study was formally implemented with predefined protocol and endpoints. |
| Participants | 7 | Lines 9-10;  Line 74  Line 89  Table 1 | Number of patients admitted in study or outbreak. Summaries of distributions of age and lengths of stays. If possible, proportion admitted from other wards, hospitals, nursing homes or from abroad. Where relevant, potential risk factors for acquiring the organism. Eligibility criteria for study. Case definitions for outbreak report. |
| Setting | 8 | Lines 66-77; supplement table2 | Description of the unit, ward or hospital and, if a hospital, the units included.  Number of beds, the presence and staffing levels of an infection control team. |
| Interventions | 9 | Lines 88-99 | Definition of phases by major change in specific infection control practice (with start and stop dates). A summary table is strongly recommended with precise details of interventions, how and when administered in each phase. |
| Culturing & Typing | 10 | na | Details of culture media, use of selective antibiotics and local and /or reference typing. Where relevant, details of environmental sampling. |
| Infection-related outcomes | 11 | Lines 73-84 | Clearly defined primary and secondary outcomes (eg incidence of infection, colonisation , bacteraemia) at regular time intervals (eg daily, weekly, monthly) rather than as totals for each phase, with at least three data points per phase and, for many two phase studies, 12 or more monthly data points per phase. Denominators (eg numbers admissions or discharges, patient bed days). If possible, prevalence of organism and incidence of colonisation on admission at same time intervals. Criteria for infection, colonisation on admission and directly attributable mortality.  For short studies or outbreak reports, use of charts with duration patient stay & dates organism detected may be useful (see text) |
| Economic outcomes | 12 | na | If a formal economic study done, definition of outcomes to be reported, description of resources used in interventions, with costs broken down to basic units, stating important assumptions. |
| Potential Threats to internal validity | 13 | Lines 104-113 | Which potential confounders were considered, recorded or adjusted for (eg: changes in length of stay, case mix, bed occupancy, staffing levels, hand-hygiene compliance, antibiotic use, strain type, processing of isolates, seasonality).  Description of measures to avoid bias including blinding & standardisation of outcome assessment & provision of care. |
| Sample size | 14 | Lines 124-127 | Details of power calculations, where appropriate |
| Statistical methods | 15 | Lines 128-144 | Description of statistical methods to compare groups or phases. Methods for any subgroup or adjusted analyses, distinguishing between planned and unplanned (exploratory) analysis. Unless outcomes are independent, statistical approaches able to account for dependencies in the outcome data should be used, adjusting, where necessary, for potential confounders.  For outbreak reports statistical analysis may be inappropriate. |
| **Results**  Recruitment | 16 | Lines 147-151  Table 1 | For relevant designs the dates defining periods of recruitment and follow-up. A flow diagram is recommended to describe participant flow in each stage of study. |
| Outcomes & estimation | 17 | Figure1 & 2  Table 2 | For the main outcomes, the estimated effect size and its precision (usually using confidence intervals). A graphical summary of the outcome data is often appropriate for dependent data (such as most time series). |
| Ancillary analyses | 18 | Figure1 & 2  Supplement tables and figures | Any subgroup analyses should be reported and it should be stated whether or not it was planned (specified in the protocol) and possible confounders adjusted for |
| Adverse events | 19 | Lines 151-153 | Pre-specified categories of adverse events and occurrences of these in each intervention group . This might include drug side effects, crude or disease specific mortality in antibiotic policy studies or opportunity costs in isolation studies. |
| **Discussion**  Interpretation | 20 | Lines 185-190 | For intervention studies an assessment of evidence for/against hypotheses, accounting for potential threats to validity of inference including regression to mean effects and reporting bias.  For outbreak reports, consider clinical significance of observations and hypotheses generated to explain them. |
| Generalisability | 21 | Lines193-201 | External validity of the findings of the intervention study i.e. to what degree can results be expected to generalise to different target populations or settings. |
| Overall evidence | 22 | Lines 202-214 | General interpretation of results in context of current evidence. |
