# Supplementary material for: Efficacy of universal preoperative decolonization with Polyhexanide in primary joint arthroplasty on surgical site infections. A multicenter before-and after-study
Source: Antimicrob Resist Infect Control. 2020 Nov 30;9:188. doi: 10.1186/s13756-020-00852-0 (PMC7708093; doi:10.1186/s13756-020-00852-0)

Supplement figure 1:

Rate of observed and modelled overall Surgical Site Infection rate (SSIR) by month of surgery in the period prior to (0-24) and after implementation of the intervention (25-48) in patients adherent to protocol and unknown adherence (other)


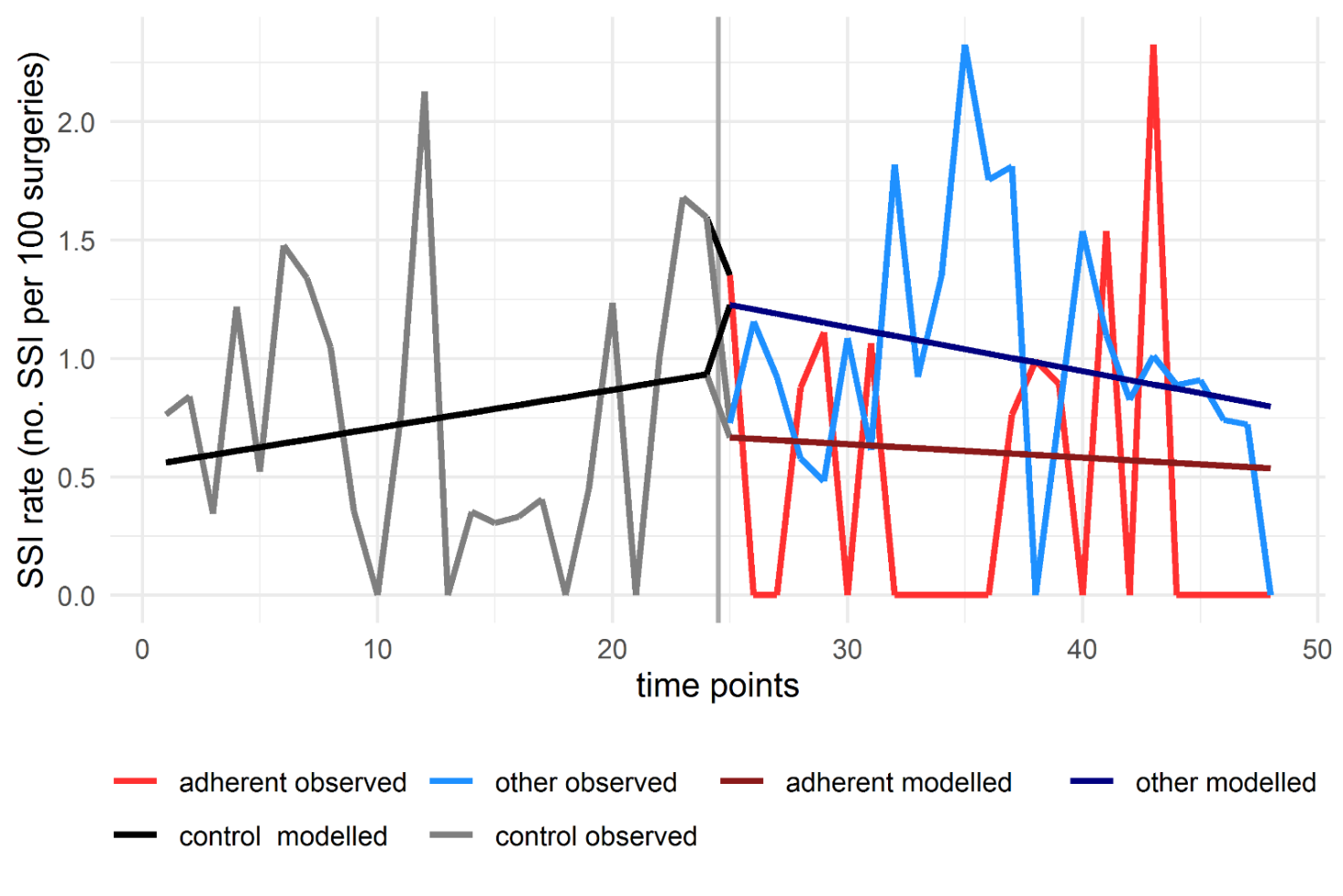

Supplement: Supplementary file 4 — Additional file 4. Supplement Figure 1. Rate of observed and modelled overall Surgical Site Infection rate (SSIR) by month of surgery in the period prior to (0–24) and after implementation of the intervention (25–48) in patients adherent to protocol and unknown adherence (other) [file 13756_2020_852_MOESM4_ESM.docx]
