# Supplementary material for: Efficacy of universal preoperative decolonization with Polyhexanide in primary joint arthroplasty on surgical site infections. A multicenter before-and after-study
Source: Antimicrob Resist Infect Control. 2020 Nov 30;9:188. doi: 10.1186/s13756-020-00852-0 (PMC7708093; doi:10.1186/s13756-020-00852-0)

Supplement figure 2:

Results of multivariable regression analysis with the endpoints overall and *S. aureus* SSI a) in all patients with elective hip arthroplasty b) in patients with elective hip arthroplasty adherent to protocol c) in all patients with elective knee arthroplasty d) in patients with elective knee arthroplasty adherent to protocol


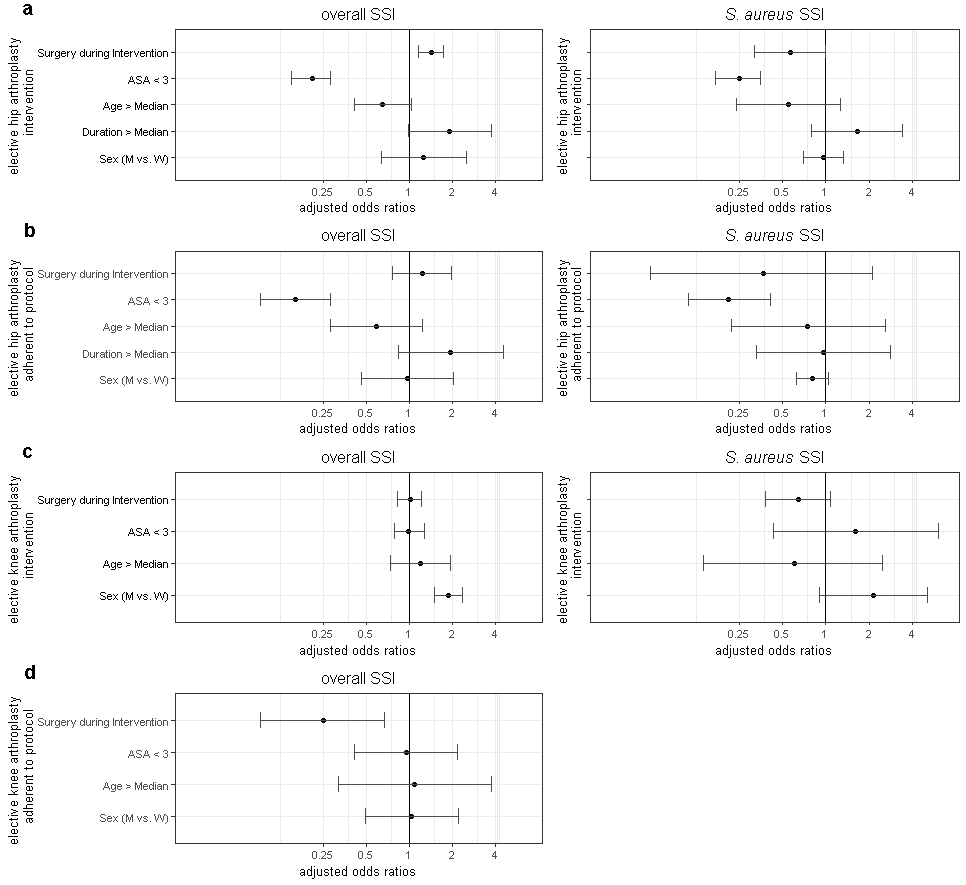

Supplement: Supplementary file 5 — Additional file 5. Supplement Figure 2. Results of multivariable regression analysis with the endpoints overall and S. aureus SSI a) in all patients with elective hip arthroplasty b) in patients with elective hip arthroplasty adherent to protocol c) in all patients with elective knee arthroplasty d) in patients with elective knee arthroplasty adherent to protocol [file 13756_2020_852_MOESM5_ESM.docx]
